# *In vitro* impact of ethanolic extract of *Bryonia laciniosa* seed on Gir bull spermatozoa: a comprehensive evaluation through transcriptome profiling

**DOI:** 10.3389/fvets.2024.1419573

**Published:** 2024-07-12

**Authors:** Jignesh Italiya, Ketankumar J. Panchal, Subhash J. Jakhesara, Chaitanya G. Joshi, Prakash G. Koringa

**Affiliations:** ^1^Department of Veterinary Biotechnology, Veterinary College, Kamdhenu University, Anand, India; ^2^Centre for Infectious Animal Diseases, Faculty of Tropical AgriSciences, Czech University of Life Sciences, Prague, Czechia; ^3^P. D. Patel Institute of Applied Sciences, Charotar University of Science and Technology, Changa, India; ^4^Department of Science and Technology, Gujarat Biotechnology Research Centre (GBRC), Government of Gujarat, Gandhinagar, India

**Keywords:** *Bryonia laciniosa*, Shivlingi, CASA, transcriptomics, bull spermatozoa

## Abstract

**Aim/objectives:**

This study examines the *in vitro* impact of an ethanolic extract derived from *Bryonia laciniosa* seeds on the Gir bull (*Bos indicus*) spermatozoa. The objective is to thoroughly assess the effects of the seed extract on the physiological parameters of bull spermatozoa, followed by evaluating its effects on X and Y-bearing spermatozoa and its impact on gene expression through transcriptome profiling.

**Material method:**

For this study, one Gir bull was selected, and 12 ejaculates were collected at one-week time intervals. Sperm cells were isolated from each ejaculate and incubated with varying concentrations of the ethanolic extract. The physiological parameters of the spermatozoa were assessed using Computer Assisted Semen Analysis (CASA) and compared with control groups to evaluate the extract’s effects on sperm quality and motility.

**Results and discussion:**

At a concentration of 18 mg/mL *B. laciniosa* extract, we noticed a statistically significant 16.4% increase in sperm motility (*p* = 0.0065). In order to understand the specific effect on X and Y-bearing spermatozoa, motile and non-motile sperm separated by glass wool column method and further evaluated for quantification of X and Y-bearing sperm in all samples by ddPCR. To understand the effect of *B. laciniosa* extract on spermatozoa at the molecular level, whole transcriptome profiling was carried out using Illumina MiSeq. Transcriptome profiling revealed 81 genes that were expressed differently between the group treated with the extract and the control group. The current investigation revealed an increase in the expression of TLX1, CRYGB, KLF13, and ZAR1 transcripts, which play a role in embryonic development. In addition, several genes have been identified that are involved in sperm motility, such GSK3B, LAPRS, MAPK1, CAMK2B, and AQP7. The findings exhibited the therapeutic effectiveness of *B. laciniosa* seeds in augmenting fertility through a synergistic blend of activities, including enhanced sperm motility and positive influence on embryogenesis.

## Introduction

The fertility of bulls, which refers to their capacity to successfully fertilize eggs, activate them, and support their further development, plays a vital role in the efficient production of cattle (1). The assessment of bull fertility is frequently disregarded when evaluating the reproductive success of a herd. Fertility features exert a substantial influence on production outcomes, rendering them highly valuable from an economic standpoint within the livestock sector. While it is uncommon for bulls to exhibit full infertility, many bulls may experience sub-fertility, which can significantly affect the overall output of a herd (2). The ability of a male bull to effectively fertilize a female bovine is contingent upon various conditions subsequent to the successful deposition of semen in the female reproductive tract. The process of fertilization involves a series of essential events, including sperm transport, sperm capacitation, and sperm attachment to the egg. Subsequently, the acrosome reaction takes place, enabling the penetration of the zona pellucida. Following this, the sperm binds and fuses with the egg plasma membrane, ultimately leading to the delivery of intact paternal genetic elements into the egg. The understanding of the various and intricate processes involved in bull fertility remains incomplete, therefore significantly constraining our capacity to accurately discern infertile and sub fertile bulls, as well as those exhibiting exceptional fertility (3–5).

Ethnomedicine has long been recognized as a crucial component in the treatment of diverse ailments throughout history. Approximately one quarter of contemporary pharmaceuticals are derived from medicinal plants that have been historically utilized (6). Ayurvedic medicine, usually referred to as Ayurveda, is a form of traditional Indian medicine that has gained recognition as a complementary and alternative approach to healthcare (7). *Bryonia laciniosa* is widely used in Ayurvedic medication, as documented in Vrishya Rasayana, an ancient Ayurvedic text (3). The plant, usually referred to as “Shivlingi,” and widely distributed across various nations including Indian sub-continent, Southeast Asia, Australia, and Tropical Africa (8). *B. laciniosa* Seeds have been used in the therapeutic management of female infertility, male infertility caused by Oligospermia, impaired spermatogenesis, Asthenozoospermia, Teratospermia. The *B. laciniosa* plant is also used for its therapeutic characteristics, encompassing analgesic, antidiabetic, antioxidant, androgenic, anticancer, and antipyretic effects (9, 10). Therefore, this particular ethnobotanical plant exhibits significant promise for further investigation in the realm of reproductive challenges pertaining to both males and females.

Recent years have seen an increase in the use of plant extracts as a natural source of compounds utilized in semen storage to protect and enhance sperm function. This is due to the fact that plants produce a wide variety of chemical compounds called phytochemicals or secondary metabolites. Several plant extracts, such as *Foeniculum vulgare* (fennel), *Phoenix dactylifera* (date palm), *Achillea millefolium* (yarrow), *Opuntia ficus-indica* (prickly pear), *Capparis spinosa* (caper bush), *Rhodiola rosea* (golden root), *Ceratonia siliqua* (carob), and others, have demonstrated the ability to enhance sperm motility and other sperm parameters when used as sperm preservatives (11). There have been few investigations on changes to sperm transcripts in response to external stimuli by *in vitro* plant derived compounds, despite the fact that sperm transcripts are abundance and there is evidence of changed gene expression in both fertile and infertile sperm (12). Moreover, a crucial subset of transcripts from early embryos is recognized to originate exclusively from sperm. The spermatozoa include a mixture of intact and biologically degraded RNAs, but their amounts are so low that traditional methods like qPCR, microarray, and hybridization techniques may not offer a comprehensive understanding of the composition and expression levels of RNA in spermatozoa. Transcriptome sequencing is a powerful technique that enables the comprehensive analysis of transcript information, including many aspects such as transcript variations, single nucleotide polymorphisms, and unique transcripts.

There is a limited amount of evidence and literature accessible about the *in vitro* impact of *B. laciniosa* extract on spermatozoa, despite its widespread use and various assertions of its effectiveness in infertility treatment. This study investigates the effects of an ethanolic seed extract of *B. laciniosa* on sperm motility using an *in vitro* approach. Furthermore, our objective is to ascertain whether there is a distinct impact on spermatozoa carrying the X or Y chromosome, along with any possible modifications in transcript abundance.

## Materials and methodology

### Preparation of S-TALP media

Sterile Sperm-Tyrode’s albumin lactate pyruvate (S-TALP) media was used as a base, containing the components outlined in Supplementary Table 1. S-TALP was prepared as described by Parrish (13), with slight modifications, and the pH was adjusted to 7.6.

### Preparation of ethanolic extract

The selected mature seeds of *B. laciniosa* were weighed at 5 g and pulverized manually using a mortar and pestle. For the defatting process, the fine powder of seeds was soaked in n-Hexane in a ratio of 1:10 (powder: n-Hexane) for 90 min at room temperature in a static condition. After that, the supernatant was aspirated, and the residual powder was allowed to dry for 2 h on a hot plate at 40°C, followed by soaking the residual powder in absolute ethanol for 24 h at room temperature in a ratio of 1:10 (powder: ethanol) with continuous shaking on the orbital rocker (Barnstead Labquake Rotisserie, IA, USA). After 24 h, the supernatant was collected in a 50-mL glass beaker and kept at 40°C on a hot plate until the complete evaporation of ethanol. After complete evaporation of ethanol, the remained dried extract was diluted in 10 mL of S-TALP media and stored at 4°C temperature for further use. It was considered a stock seed extract.

### Semen collections and evaluation

The current investigation was conducted on a semen sample obtained from a one Gir bull (*Bos indicus*). Fresh semen samples were obtained from one Gir bull at the Central Semen Station, which is part of the Department of Animal Reproduction Gynecology & Obstetrics at the College of Veterinary Science & Animal Husbandry in Anand. Total 12 ejaculation were collected during this study at 1 week interval from same bull. The samples were collected during the morning period from 7:30 to 8:30 am. The semen samples were obtained by collecting ejaculates using an artificial vagina positioned over a model buffalo bull. Following collection, the semen-containing tubes were promptly immersed in a water bath at a constant temperature of 34°C. Subsequently, the samples were assessed for a range of macroscopic and microscopic seminal attributes. The initial five ejaculates were utilized to examine the effects of various concentrations of the plant extract on sperm motility using microscope. Following, seven ejaculations were used for further analysis by CASA and subsequent experiments.

### Somatic cell removal and sperm count

The semen sample was transferred to a 15 mL centrifuge tube and centrifuged at 1,500 revolutions per minute for 5 min at ambient temperature. The resulting pellet of sperm was subsequently re-suspended in 3 mL of S-TALP medium. Sperm count was determined using a hemocytometer in accordance with the established protocol for assessing sperm count.

### Treatment of seed extract to sperm

The stock seed extract (40 mg/mL) was used to prepare five different concentrations in S-TALP medium. The seed extract concentrations were selected based on two-fold dilution series (2, 4, 8, 16, and 32) along with stock extract. In addition to the aforementioned five doses of seed extract, a control was included in all trials, consisting of 1 mL of S-TALP and 1 mL of 1× PBS. Diluted sperm pellets from each ejaculates were resuspended in S-TALP medium. A total of 0.1 million spermatozoa (0.5–2 μL, depending on the sperm count per ejaculate) suspended in S-TALP medium were added to distinct concentrations of seed extracts in individual 2 mL microfuge tubes. The samples were then incubated in a CO_2_ incubator at 37°C for 1 h. The initial five ejaculates were utilized to examine the effects of various concentrations of the plant extract on sperm motility using microscope. Based on the initial results, extract concentrations of 16 and 18 mg/mL were selected for further examination of changes in the physiological parameters of spermatozoa after treatment, using the CASA system. Further statistical analyses were performed using R (version 4.3).

### Separation of motile and non-motile sperm

The mobile sperm cells were isolated using a glass-wool column, following the protocol described by Van der Ven et al. (14). The motile sperm was effectively able to traverse through a glass wool barrier and was subsequently collected in a 1.5 mL microfuge tube for the purpose of conducting quantification of X and Y-bearing spermatozoa and transcriptome profiling. The column contained immobile and non-viable spermatozoa, which were subsequently collected for quantification of X and Y-bearing spermatozoa and transcriptome analysis. The DNA was isolated from distinct motile and non-motile spermatozoa by using a DNA extraction kit (Qiagen, USA). The assessment of DNA quality was conducted using the NanoDrop 1000 Spectrophotometer manufactured (Thermo Scientific, USA).

### Quantification of X and Y-bearing spermatozoa

The quantification of X and Y-bearing spermatozoa was conducted by employing digital droplet PCR (ddPCR) on both motile and non-motile spermatozoa collected by sperm separation method after treatment. The development of TaqMan hydrolysis probes for the amplification of SRY (Y chromosome-specific) and F9 (X chromosome-specific) gene target sequences in ddPCR was conducted in accordance with the directions provided by the manufacturer. FAM/VIC fluorescent dye combinations were used for the purposes of absolute quantification and the development of duplex assays.

### Transcriptome profiling of seed extract treated spermatozoa

The concentration that yielded the highest spermatozoa motility was chosen for transcriptome profiling. Four distinct samples have been chosen for transcriptome profiling, comprising of a control sample of moving spermatozoa, a control sample of non-moving spermatozoa, a sample of treated motile spermatozoa, and a sample of non-motile spermatozoa. The four samples underwent centrifugation at a speed of 2,500 revolutions per minute for a duration of 6 min at a temperature of 34°C, resulting in the formation of pellets. Each pallet was diluted in 1× PBS, with each 7 μL sample containing 600 sperms, for subsequent utilization. Amplification of RNA (Poly A+ mRNA) from a single cell, enzymatic fragmentation, and library preparation was carried out according to the manufacturer’s protocol using QIAseq FX Single Cell RNA Library Kit (Qiagen, Germany). Each library was prepared using a total of 1,000 ng of diluted DNA. Each DNA amplicon library was normalized to 2 nM for amplicon sequencing with an average of 8 pM with 2% phiX control on the MiSeq platform (Illumina, USA) at Anand Agricultural University, Anand according to the manufacturer’s instructions.

### Bioinformatics analysis

STARaligner v2.6 was used to map sequences to *Bos taurus* genome build 4.6.1. The differentially expressed genes (DEGs) between groups were discovered using the ‘Cuffdiff’ module, with a requirement of an absolute log2 fold change more than 2 between distinct groups and a false discovery rate (FDR) of *p* < 0.05. Following differential gene expression analysis, gene ontology was performed using DAVID v6.8 on frequently expressed highly abundant genes. The data was analyzed using Microsoft excel. One and two-way ANOVA followed by DUNNETs multiple comparison tests were used to test for significant differences among the individual treatment combinations.

## Results

### Macroscopic and microscopic features of semen

The average sperm concentration of semen observed in the studied Gir cattle bulls was 1111.57 ± 29.46 million sperm per milliliter. The sperm concentration per unit volume is crucial in semen processing since the dilution rate depends on the concentration of spermatozoa in each ejaculation, together with their initial motility and viability. The semen color in Gir bulls was determined to be milky white to yellowish, which is the typical color for Gir cattle bull semen. This indicates that the bulls’ genital tracts were healthy and free from any illness or damage, and the ejaculates were uncontaminated.

#### Microscopic evaluation of spermatozoa following treatment with seed extract

The mean mass activity scores (rated on a scale of 0–5), as well as the percentages of initially motile and live sperms in the semen of Gir cattle, were found to be 3.35 ± 0.08, 81.50 ± 1.30%, and 83.70 ± 1.04%, respectively. Individual sperm motility and viability are crucial factors in assessing semen quality and can provide valuable insights into the potential of the semen for advanced processing. Upon initial examination under a microscope, it was observed that sperm motility and viability were higher in sperms treated with 16 mg/mL of extract (as shown in Table 1). Therefore, to conduct a more detailed evaluation of the effects of the seed extract on the physiological parameters of spermatozoa after treatment, two concentrations were selected: 16 mg/mL and 18 mg/mL.

**Table 1:** Sperm motility and live sperm percentage under effects of seed extract with a comparison of other control groups.

No.	Shivlingi seed extract treated group	Live sperm (Percentage) (*n* = 5)	Motility (0–5 scale) (*n* = 5)
1	S-TALP (1,000 μL)	67.5 ± 6.4	2.62 ± 0.47
2	2 mg/mL	65 ± 7.0	2.1 ± 0.25
3	4 mg/mL	63.75 ± 4.78	2 ± 0
4	8 mg/mL	65 ± 4.08	2.75 ± 0.28
5	16 mg/mL	75 ± 4.08	3.37 ± 0.47
6	32 mg/mL	51.25 ± 14.36	2.5 ± 0.57
7	40 mg/mL	27.5 ± 6.45	2 ± 0.81
8	1× PBS (1,000 μL)	67.5 ± 2.8	2.62 ± 0.75

#### Evaluation of spermatozoa after treatment of seed extract by CASA

**Figure 1:**
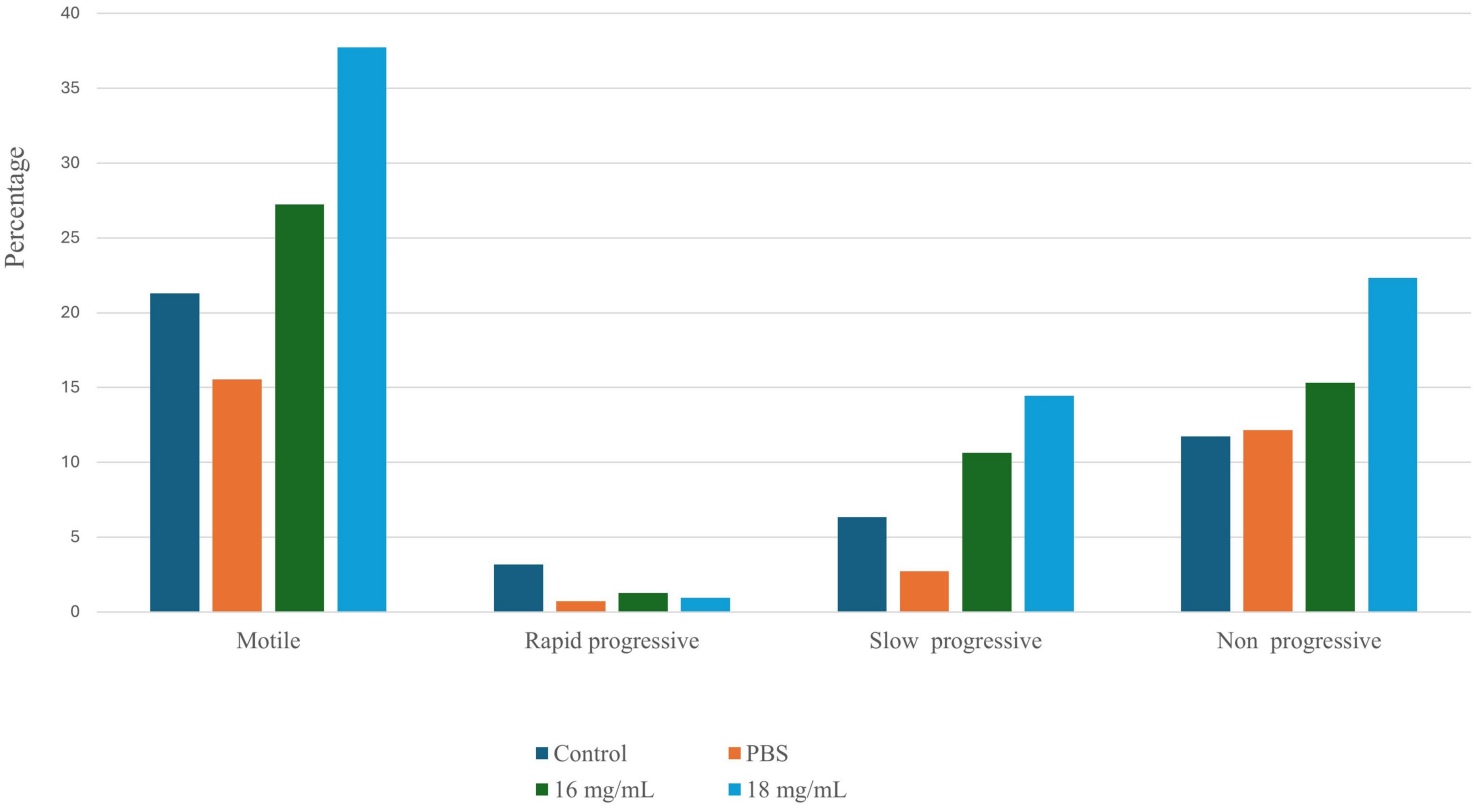
The sperm motility profile was assessed under various conditions using the Biovis CASA system.

The sperm motility profile of spermatozoa treated with seed extract was promptly examined using the Biovis CASA system (Expert Vision labs Pvt. Ltd., Mumbai, India). Sperm treated with a seed extract concentration of 18 mg/mL demonstrated a significant increase in motility (*p* < 0.05). Figure 1 illustrates the comparative sperm motility profile of control and treatment group. In addition to an elevation in spermatozoa motility, there was a substantial rise in the proportion of non-progressive and slow-progressive spermatozoa in the group treated with the plant extract. The proportion of rapidly progressing spermatozoa varies depending on the state of the sample and is greater when treated with Seed extract at a dosage of 18 mg/mL. Based on the aforementioned observation, two distinct doses have been chosen for subsequent studies.

#### Statistical analysis

The Shapiro–Wilk normality test was applied to determine if the dataset was normally distributed. The results indicated that both the control group (*p*-value = 0.7631) and the 16 mg/mL treatment group (*p*-value = 0.6633) were normally distributed. However, the 18 mg/mL treatment group was not normally distributed (*p*-value = 0.002063). Consequently, non-parametric methods, including the Kruskal–Wallis test and pairwise Wilcoxon tests, were utilized for group comparisons. The results suggest a significant 16.4% increase in sperm motility in the 18 mg/mL treatment group (*p*-value = 0.0065).

### Assessment of sperm velocity and kinematics

The CASA study of freshly treated spermatozoa provided information on the mean sperm velocity and kinematic attributes. The CASA analysis revealed a notable reduction in various kinematic parameters of the treated spermatozoa, including Average Path Velocity, Curvilinear Velocity, Straight Line Velocity, Straightness, Wobbling Index, Beat-Cross Frequency, and Dancing (as shown in Table 2), when compared to the normal spermatozoa samples from Gir bulls (15).

**Table 2:** Mean (±SE) spermatozoa kinematics assessment with comparison of treatment group spermatozoa and other controls.

Seminal attribute	Normal value	Control (*n* = 7)	1× PBS (*n* = 7)	16 mg/mL (*n* = 7)	18 mg/mL (*n* = 6)
Average path velocity (VAP, μm/s)	50.01 ± 1.25	41.71 ± 4.7	34.77 ± 1.9	28.74 ± 2.2	28.43 ± 2.5
Curvilinear velocity (VCL, μm/s)	88.62 ± 1.66	89.22 ± 6.3	79.62 ± 5.6	65.51 ± 3.6	66.66 ± 5.8
Straight line velocity (VSL, μm/s)	44.51 ± 1.35	38.97 ± 4.9	32.02 ± 2.1	26.68 ± 2.3	25.63 ± 2.6
Linearity (LIN, %)	50.06 ± 1.42	38.54 ± 7.2	40.17 ± 1.4	40.72 ± 2.8	40.26 ± 3.5
Straightness (STR, %)	85.17 ± 0.92	92.14 ± 2.2	91 ± 1.7	91.84 ± 1.3	88.95 ± 1.5
Wobbling index (WOB, %)	57.00 ± 1.17	46.85 ± 4.2	43.82 ± 1.3	43.9 ± 2.5	45.08 ± 3.2
Beat-cross frequency (BCF, Hz)	15.55 ± 0.58	12.64 ± 1.5	11.15 ± 1.5	8.22 ± 1.8	8.18 ± 1.2
Lateral head displacement (ALH, μm)	2.39 ± 0.18	3.01 ± 0.2	2.85 ± 0.3	2.65 ± 0.1	2.45 ± 0.2
Dancing velocity (DNC, μm^2^/s)	208 ± 15.5	240 ± 26.1	201.5 ± 31.8	154.6 ± 12.5	156.4 ± 30.7
Dancing mean (DNM, μm^2^/s)	5.70 ± 0.46	6.95 ± 1.2	6.4 ± 0.8	6.11 ± 0.4	11.26 ± 4.6

### Quantification of X and Y spermatozoa

**Figure 2:**
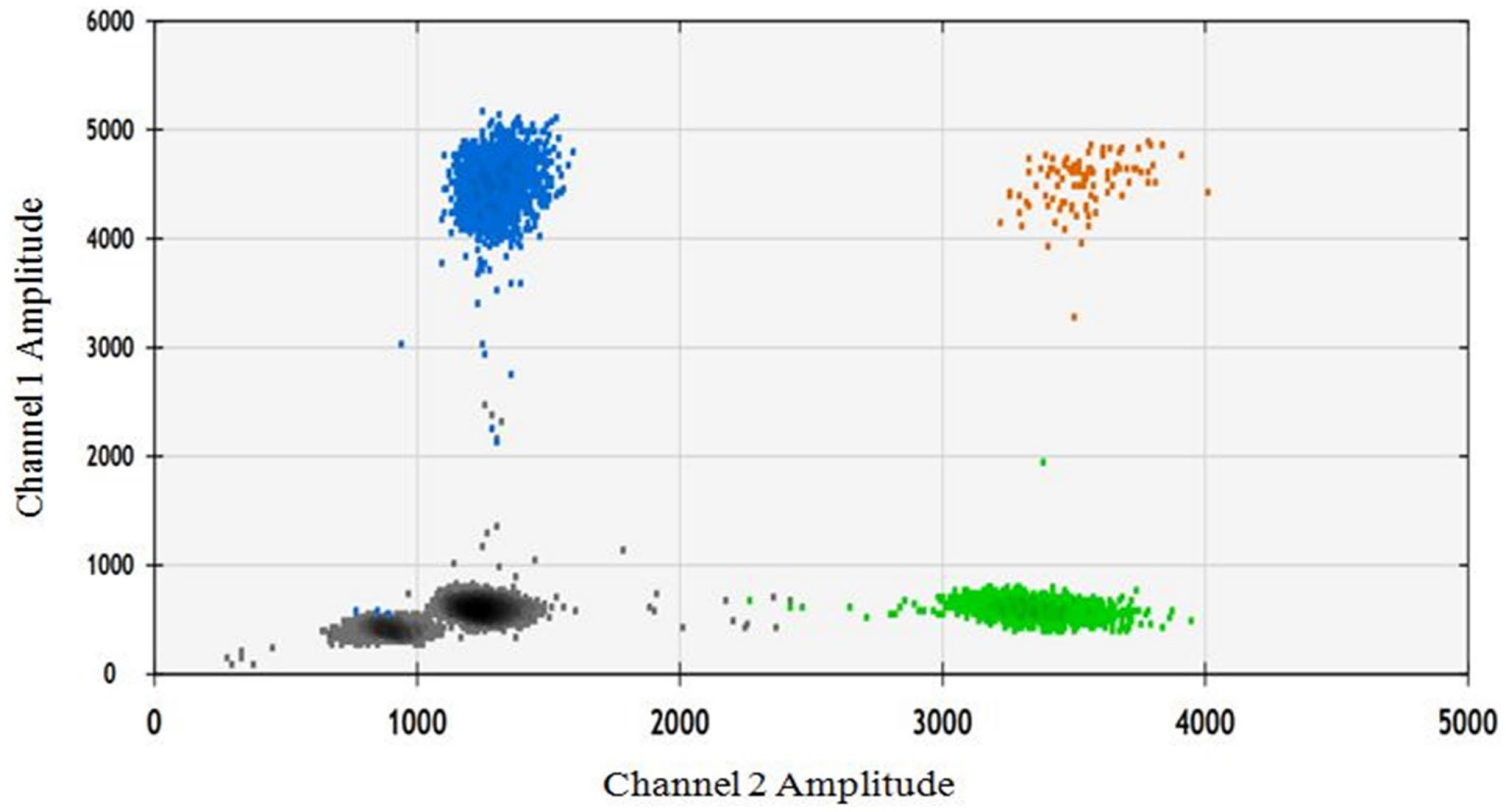
The ddPCR 2D fluorescence plot depicts two amplicons: the gray color represents the double negative, the blue color represents the FAM positive/F9 target, the green color represents the VIC positive, and the brown color represents the double positive.

In order to assess the impact of seed extract on spermatozoa carrying the X and Y chromosomes, we isolated motile (live) and non-motile spermatozoa from both the control and treatment groups using the glass wool column technique. Out of the two treatment groups (16 and 18 mg/mL), the 18 mg/mL group was selected as it exhibited higher motility compared to the other group (Figure 1). The quantity and proportion of targets for each sample are detailed in Table 3. The results for F9 (X chromosome-specific) and SRY (Y chromosome-specific) did not demonstrate any statistically significant difference in the percentage of motile and non-motile sperm (Figure 2).

**Table 3:** F9 and SRY count from each sample.

Sr. no.	Sample ID	F9	Percentage	SRY	Percentage
1	NTC (no template control)	No call	0%	No call	0%
2	Control S-TALP (motile sperm)	70	50%	70	50%
3	Control S-TALP (non-motile sperm)	33.6	49.77%	33.9	50.23%
4	1× PBS (motile sperm)	43	51.86%	39.9	48.14%
5	18 mg/mL (motile sperm)	30.5	50.16%	30.7	49.84%
6	18 mg/mL (non-motile sperm)	42	50.11%	41.8	49.89%

### Transcriptome profiling of seed extract treated spermatozoa

**Figure 3:**
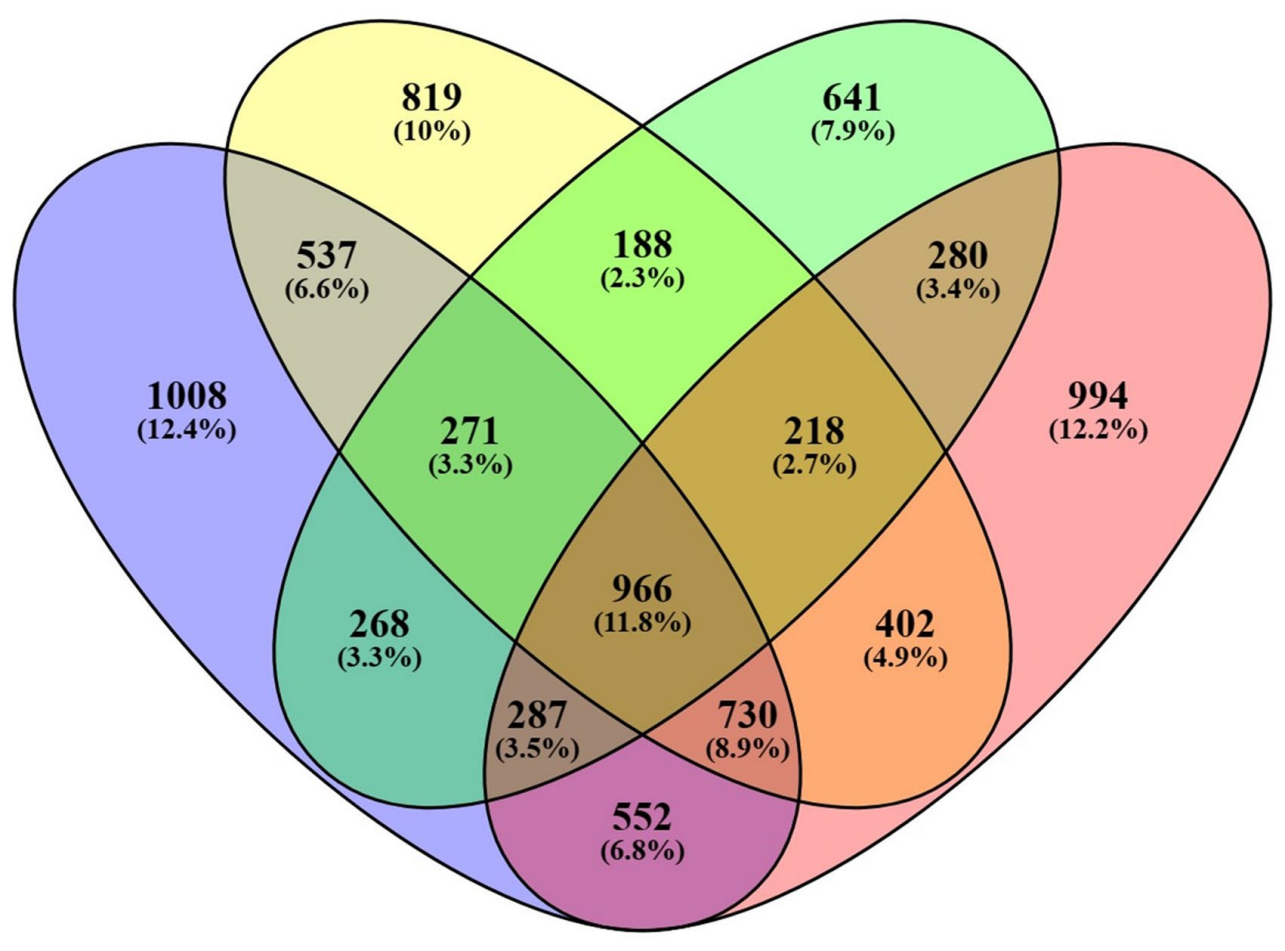
Venn diagrams illustrating the number of genes expressed with FPKM values greater than zero in all four samples, namely control motile, control non-motile, Shivlingi treated motile, and Shivlingi treated non-motile.

The treatment group, with a concentration of 18 mg/mL, was selected in order to assess the impact of seed extract on the transcriptome profile of spermatozoa, in comparison to the control group. The analysis of the control and treatment group data reveals the existence of nearly 2 million RNA sequence reads, each with an average length of around 400 base pairs (detailed in Supplementary Table 2). RNA sequencing analysis revealed that 4,142 genes were expressed with Fragments Per Kilobase Million (FPKM) greater than zero in the control motile sample, whereas 4,632 genes were expressed in the sample of motile spermatozoa treated with *B. laciniosa*. In the control non-motile group, a total of 3,122 genes were expressed. However, in the *B. laciniosa* treated non-motile group, a higher number of genes, namely 4,445, were expressed with FPKM > 0. This information is illustrated in Figure 3.

### Differential gene expression of control and treatment group spermatozoa

**Figure 4:**
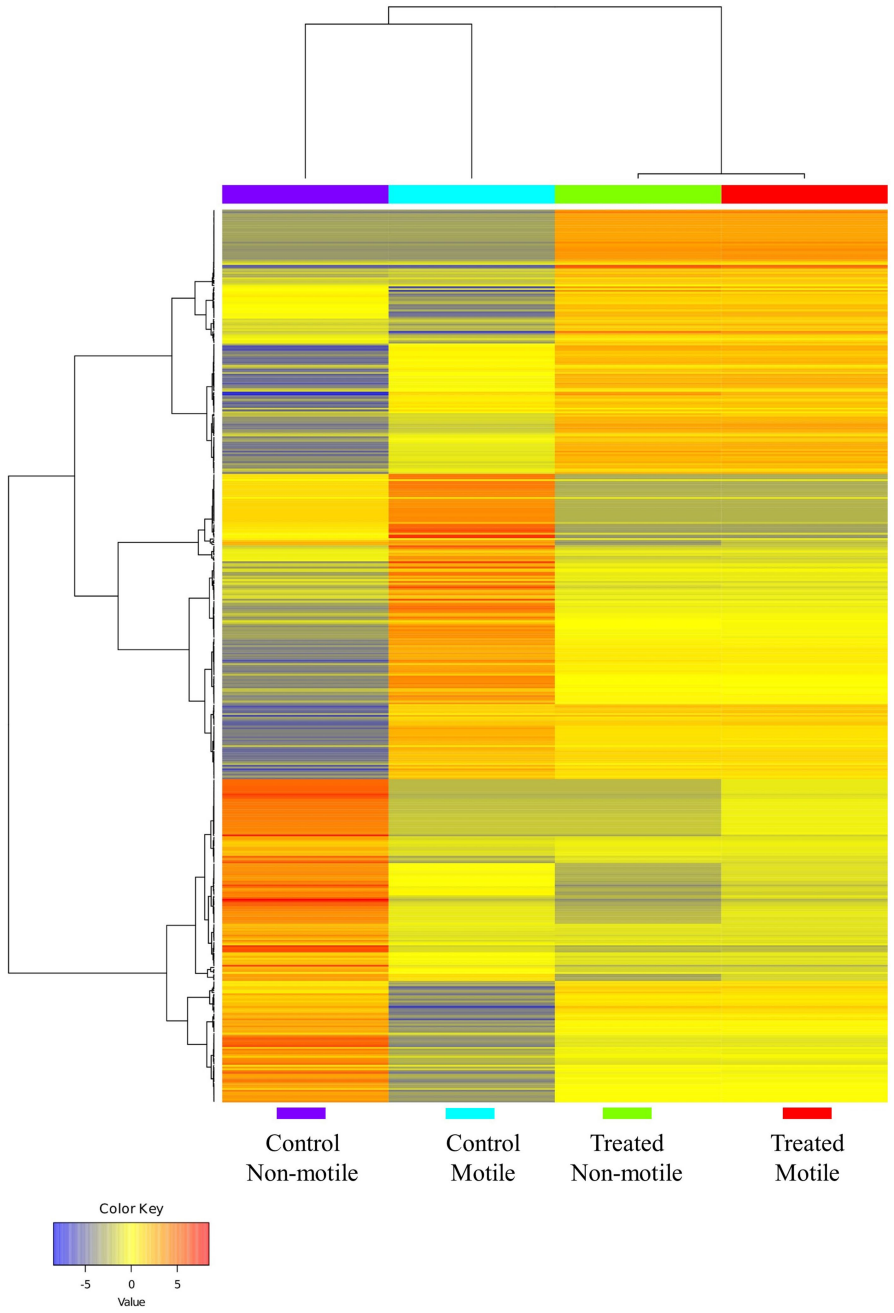
The heatmap illustrates the clustering of genes and sample groups based on their expression levels (generated by using ShinyGO 0.77).

Differential expression analysis was performed using Cufflinks suits (version 2.2.1), and the normalized gene expression value was computed as fragments per kilobase of transcript per million mapped. In comparison to the control motile spermatozoa sample, the expression of 43 genes was significantly different in the *B. laciniosa* treated motile spermatozoa. A total of 24 genes exhibited upregulation, whereas 19 genes demonstrated downregulation. The genes that are most significantly expressed are illustrated in Supplementary Table 3. In comparison to the control non-motile spermatozoa sample, the expression of 38 genes was significantly different in non-motile spermatozoa treated with *B. laciniosa*. Twenty genes exhibited upregulation, whereas 18 genes demonstrated downregulation. The genes that are most significantly expressed are illustrated in Supplementary Table 4. The heatmap demonstrates an increase in gene expression in the control motile and control non-motile groups compared to the treatment motile and treated non-motile groups, which are clustered together as shown in Figure 4.

### Functional annotation and pathway analysis

**Figure 5:**
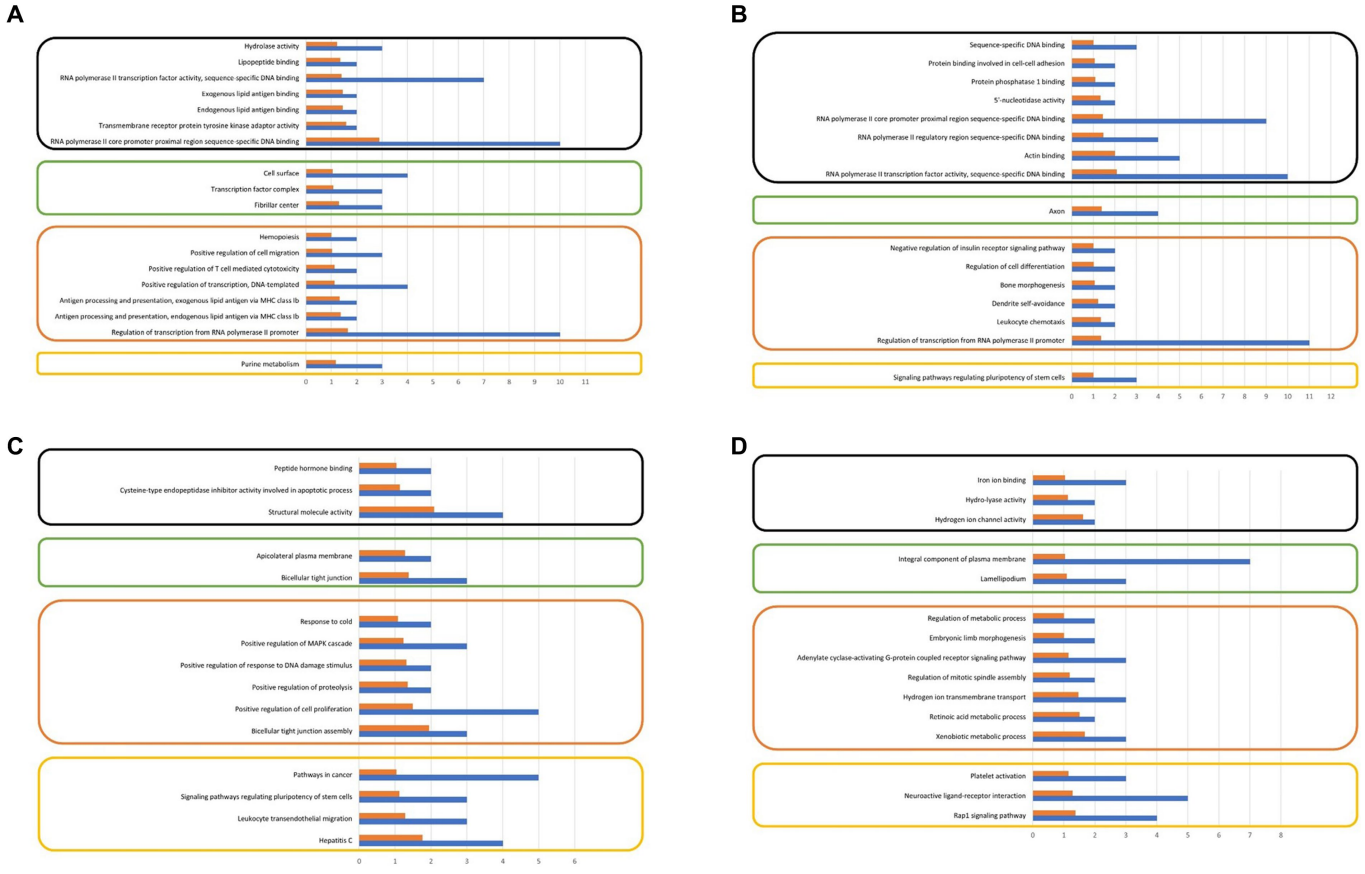
**(A)** Gene ontology analysis of significantly expressing genes at control motile, **(B)** Gene ontology analysis of significantly expressing genes at treated motile, **(C)** Gene ontology analysis of significantly expressing genes at control non-motile, **(D)** Gene ontology analysis of significantly expressing genes at treated non-motile (FPKM ≥ 500).

The analysis of gene ontologies for significant genes (FPKM > 500) across all control and treatment groups identified the KEGG pathway, molecular function, and biological process, are illustrated in Figure 5. The gene ontology analysis revealed biological processes that may potentially be associated with sperm motility and play a significant role in embryonic development.

## Discussion

Herb Shivlingi (*B. laciniosa*) has been acclimated traditionally in India as a tonic, anti-inflammatory, and astringent. Additionally, it exhibits efficacy in the treatment of inflammations, general weakness and the seeds possess curative properties for sterility (16). The seeds of *B. laciniosa* are a key component of the Ayurvedic composition ‘Strirativallabhpugpak’ mentioned in ancient literature, which is said to enhance sexual behavior and serve as a general tonic (17). The study conducted on male rats documented that the oral administration of an ethanolic extract of *B. laciniosa* resulted in substantial rise in sperm count in the epididymis and fructose content in the seminal vesicle. The application of the extract therapy resulted in a notable elevation in blood testosterone and luteinizing hormone levels (18).

The current investigation showcased that the ethanolic extract of dehydrated *B. laciniosa* seed (18 mg/mL) had significant benefits for enhancing the overall motile sperm count as well as non-progressive and slow-progressive sperm cells in an *in vitro* environment. Our investigation revealed that the macroscopic and microscopic inspection of Gir bull spermatozoa was confirmed to be normal, as described by Pathak et al. (15). Microscopic analysis conducted using the CASA system indicated a minor reduction in VAP, VCL, VSL, and linearity of *B. laciniosa* treated spermatozoa, which might impact male fertility. Nevertheless, no substantial disparity was seen when compared to the 1× PBS control group. To comprehend the cytotoxic impact of the ethanolic extract on X and Y-bearing spermatozoa, we conducted absolute quantification of the treated and control spermatozoa groups following their separation using the glass wool column technique. The measurement by ddPCR did not detect any significant alterations in the proportion of X and Y-bearing spermatozoa in both motile and non-motile spermatozoa from the control and treatment groups.

The transcriptome analysis revealed an approximate expression profile of 3.5–4.5 thousand genes across all control and treatment groups. The majority of the numerous spermatozoa transcripts were intact. This diverse RNA population plays crucial roles in the packing of paternal chromatin, the maturation and capacitation of sperm, fertilization, early embryogenesis, and the maintenance of development. Our research findings indicate that there is a notable presence of spermatozoan transcripts related to biological processes, cellular components, and molecular activities in both the control and treatment groups, as demonstrated by ontology analysis. The following biological process pathways have been identified: the construction of bicellular tight junctions; the positive regulation of cell proliferation; the positive regulation of proteolysis; the positive regulation of response to DNA damage stimuli; and the positive regulation of the MAPK cascade in the treated motile spermatozoa group. In the case of the control group, this includes the xenobiotic metabolic process, the retinoic acid metabolic process, hydrogen ion transmembrane transport, and the regulation of mitotic spindle assembly observed.

Differential gene expression analysis revealed that motile spermatozoa treated with *B. laciniosa* extract exhibited significant differential expression of TLX1, CRYGB, KLF13, and ZAR1genes, which are involved in embryo development. Additionally, other genes such as KLF13, RPL32, STOML3, CLDN15, PRDX3, SELENOM, CAMK2B, MAPK1, INSM2, ARF4, NAGK, AQP7, GPX6, and GJC3 also showed significant differential expressions. We found that the expression of several genes, including ZAR1, NOL7, MAPK1, ARF4, AQP7, MPC1, CHMP4C, IL16, and FRMD3, was increased in motile spermatozoa treated with *B. laciniosa* extract. Interestingly, some of these genes were also shown to be elevated in a recent work that evaluated the transcriptome profile of spermatozoa treated with an ethanolic seed extract of Putranjiva (*Putranjiva roxburghii*) *in-vitro* (19). A recent study on the effects of Delta-9-tetrahydrocannabinol (THC) on spermatozoa demonstrated alterations of gene expression (12).

Despite the absence of cytoplasmic ribosomes, sperm are typically considered to be translationally inactive. However, additional investigation indicates that some transcripts in fully developed sperm are actively translated by mitochondrial ribosomes during capacitation (20, 21). In our investigation, we discovered an upregulation of ZAR1 in the treated motile group. ZAR1 is the initial oocyte-specific maternal-effect gene that operates throughout the transition from oocyte to embryo. The ZAR1 transcript derived from spermatozoa likely has a direct function in the development of oocytes into embryos (22). This discovery provides fresh understanding of the commencement of embryonic development and the regulation of fertility in animals. KLF13 was observed to be upregulated, exhibiting widespread expression in several biological tissues. It functions in promoting cell proliferation, cell differentiation, cell cycle progression, hematopoietic development, and inflammatory response (23).

The treatment group had an increase in the expression of GSK3B. The latest study indicates that GSK3B (Glycogen Synthase Kinase-3) has a role in regulating sperm motility and acrosome reaction in goats by influencing energy metabolism (24). The treatment group exhibited a significant increase in the abundance of the LPAR5 gene, which is also present in ejaculated human spermatozoa. This gene plays a crucial role in maintaining sperm viability through active LPA (Lysophosphatidic acid) signaling. The regulation of this signaling pathway is primarily controlled by PKC (Protein kinase C), with a smaller contribution from RT-PTK (receptor-type tyrosine-protein kinase) (25). Activation of LPAR5 by LPA stimulates the activation of G proteins, which in turn boosts adenylyl cyclase activities. This leads to changes in intracellular signaling molecules, including the buildup of cAMP and the levels of intracellular calcium. As a result, it regulates many cell biological responses. The data indicates that LPA enhanced the VCL and ALH characteristics of sperm, indicating that LPA may serve as a possible stimulant during the contact between sperm and egg (26). The signaling of lysophosphatidic acid (LPA) is crucial for preserving the survival of germ cells during the process of spermatogenesis in mice (25). MAPK1 was found down regulated in treatment group which have role in inhibition of sperm capacitation and are involved in the regulation of capacitation (27). In human spermatozoa it has been observed that an increased expression of MAPK1 (also known as ESR1: extracellular signal-regulated kinases 1) and activated p38 can predict poor human sperm quality.

On the other hand, in our treatment motile group we observed a decrease in the expression of CAMK2B. The CAMK2B protein plays a role in the Ca (2+)/CaM/CaMKII signaling pathway in the sperm main component, which is responsible for controlling sperm motility (28). We have noticed that the decrease in expression of the AQP7 gene in the treatment group may have an impact on sperm motility. AQP7 exhibits a specific distribution in the testis of humans. AQP7 likely has a role in preserving sperm motility. Moreover, the absence of AQP7 expression in sperm might potentially serve as a fundamental cause of male infertility (29). The ATP1A4 was seen to be elevated during the process of bovine sperm capacitation, namely through translation in mitochondrial ribosomes (21). Our observation indicates that the control motile group exhibited the greatest expression of ATP1A4 (FPKM = 217) compared to the treatment group. This suggests that more investigation is needed to understand the role of capacitation in the treatment group treated with ethanolic seed extract.

The research has several limitations, one of which is the lack of experimental validation using RT-qPCR. This might introduce uncertainties regarding the repeatability of the gene expression findings. The seeds of *B. laciniosa* include phytochemicals such as punicic acid, goniothalamine, and glucomannan. However, this study does not include a phytochemical examination of the ethanolic extract derived from dried *B. laciniosa* seeds. In this investigation, meticulous separation of somatic cells and seminal fluid was conducted; yet the potential for contamination of the seminal fluid should not be disregarded.

## Data availability statement

The datasets presented in this study can be found in online repositories. The names of the repository/repositories and accession number(s) can be found in the article/Supplementary material.

## Ethics statement

The animal study was approved by College of Veterinary Science and Animal Husbandry, Anand. The study was conducted in accordance with the local legislation and institutional requirements.

## Author contributions

JI: Data curation, Formal analysis, Investigation, Methodology, Software, Visualization, Writing – original draft, Writing – review & editing. KP: Data curation, Formal analysis, Software, Validation, Writing – review & editing. SJ: Conceptualization, Investigation, Resources, Supervision, Validation, Writing – review & editing. CJ: Conceptualization, Investigation, Resources, Supervision, Visualization, Writing – review & editing. PK: Conceptualization, Funding acquisition, Investigation, Project administration, Resources, Software, Supervision, Visualization, Writing – review & editing.

## Supplementary material

Supplementary Data Sheet 1 Differential gene expression between control motile and seed-S treated motile spermatozoa.

Supplementary Data Sheet 2 Differential gene expression between control non-motile and seed-S treated non-motile spermatozoa.

Supplementary Data Sheet 3 Gene ontology analysis of significantly expressing genes at Control motile, Control non-motile, treated motile, and treated non-motile.

Supplementary Data Sheet 4 Detailed spermatozoa kinematics assessment of all treated and non-treated samples used in this study.

Supplementary Data Sheet 5 Detailed statistical analysis used in this study.
