# Supplementary material for: In vitro impact of ethanolic extract of Bryonia laciniosa seed on Gir bull spermatozoa: a comprehensive evaluation through transcriptome profiling
Source: Front Vet Sci. 2024 Jul 12;11:1419573. doi: 10.3389/fvets.2024.1419573 (PMC11273328; doi:10.3389/fvets.2024.1419573)
Supplement: Supplementary Data Sheet 5 — Detailed statistical analysis used in this study. [file Data_Sheet_5.docx]

Data

| Samples | Control | PBS | Low | High |
| --- | --- | --- | --- | --- |
| A | 17.6 | 12.3 | 32.2 | 33.9 |
| B | 10.2 | 13.3 | 19.7 | 32.6 |
| C | 20.8 | 13.3 | 28.8 | 31.6 |
| D | 28.8 | 15.2 | 28.3 | 64.3 |
| E | 23.7 | 23.5 | 25 | 28.8 |
| F | 26.7 | 19 | 30 | 36 |
| G | 21.2 | 12.2 | 26.6 | 37 |

Long_imp2

> print(long_imp2)

Samples Group Abundance

1 A Control 17.6

2 B Control 10.2

3 C Control 20.8

4 D Control 28.8

5 E Control 23.7

6 F Control 26.7

7 G Control 21.2

8 A PBS 12.3

9 B PBS 13.3

10 C PBS 13.3

11 D PBS 15.2

12 E PBS 23.5

13 F PBS 19.0

14 G PBS 12.2

15 A Low 32.2

16 B Low 19.7

17 C Low 28.8

18 D Low 28.3

19 E Low 25.0

20 F Low 30.0

21 G Low 26.6

22 A High 33.9

23 B High 32.6

24 C High 31.6

25 D High 64.3

26 E High 28.8

27 F High 36.0

28 G High 37.0

Check normality with Shapiro Test

> print(shapiro_test_control)

Shapiro-Wilk normality test

data: long_imp2$Abundance[long_imp2$Group == "Control"]

W = 0.95369, p-value = 0.7631

> print(shapiro_test_pbs)

Shapiro-Wilk normality test

data: long_imp2$Abundance[long_imp2$Group == "PBS"]

W = 0.81415, p-value = 0.05638

> print(shapiro_test_low)

Shapiro-Wilk normality test

data: long_imp2$Abundance[long_imp2$Group == "Low"]

W = 0.94271, p-value = 0.6633

> print(shapiro_test_high)

Shapiro-Wilk normality test

data: long_imp2$Abundance[long_imp2$Group == "High"]

W = 0.67689, p-value = 0.002063

Based on the results of the Shapiro-Wilk normality tests:

- Control and Low groups have p-values greater than 0.05, indicating that these groups are normally distributed.
- PBS group has a p-value slightly greater than 0.05, which suggests it might be normally distributed, but it's borderline.
- High group has a p-value less than 0.05, indicating that this group is not normally distributed.

Given these mixed results, the data does not meet the assumption of normality for all groups. Therefore, it is more appropriate to use non-parametric methods such as the Kruskal-Wallis test and pairwise Wilcoxon tests for the group comparisons.

> kruskal_result <- kruskal.test(Abundance ~ Group, data = long_imp2)

> print(kruskal_result)

Kruskal-Wallis rank sum test

data: Abundance by Group

Kruskal-Wallis chi-squared = 20.052, df = 3, p-value = 0.0001656

> # Perform Pairwise Wilcoxon Tests with Benjamini-Hochberg correction

> pairwise_wilcox <- pairwise.wilcox.test(long_imp2$Abundance, long_imp2$Group, p.adjust.method = "BH")

> print(pairwise_wilcox)

Pairwise comparisons using Wilcoxon rank sum test with continuity correction

data: long_imp2$Abundance and long_imp2$Group

Control PBS Low

PBS 0.0963 - -

Low 0.0963 0.0065 -

High 0.0065 0.0065 0.0131

The Kruskal-Wallis test has a p-value of 0.0001656, which is much lower than the significance threshold of 0.05. This suggests that there are significant differences in abundance among at least some of the groups.

The pairwise Wilcoxon test results with Benjamini-Hochberg correction show the following p-values:

- **Control vs. PBS**: p = 0.0963
- **Control vs. Low**: p = 0.0963
- **Control vs. High**: p = 0.0065 (significant)
- **PBS vs. Low**: p = 0.0065 (significant)
- **PBS vs. High**: p = 0.0065 (significant)
- **Low vs. High**: p = 0.0131 (significant)

**Interpretation:**

- **Control vs. PBS**: Not significant (p = 0.0963)
- **Control vs. Low**: Not significant (p = 0.0963)
- **Control vs. High**: Significant (p = 0.0065)
- **PBS vs. Low**: Significant (p = 0.0065)
- **PBS vs. High**: Significant (p = 0.0065)
- **Low vs. High**: Significant (p = 0.0131)

These results suggest that:

- There are significant differences between the Control and High groups.
- There are significant differences between the PBS and Low groups.
- There are significant differences between the PBS and High groups.
- There are significant differences between the Low and High groups.

However, there are no significant differences between the Control and PBS groups or between the Control and Low groups.
