# Supplementary material for: In vitro impact of ethanolic extract of Bryonia laciniosa seed on Gir bull spermatozoa: a comprehensive evaluation through transcriptome profiling
Source: Front Vet Sci. 2024 Jul 12;11:1419573. doi: 10.3389/fvets.2024.1419573 (PMC11273328; doi:10.3389/fvets.2024.1419573)
Supplement: Supplementary file 6 [file Table_1.docx]

**Table 1: Composition of S-TALP media**

| **No.** | **Component** | **Conc. (mM)** | **Weight** |
| --- | --- | --- | --- |
| 1 | Sodium pyruvate (C_3_H_3_NaO_3_) | 1 mM | 44.0 mg |
| 2 | Sodium L-lactate (C_3_H_5_NaO_3_) | 3.1 mM | 560.0 mg |
| 3 | Dextrose (C_6_H_12_O_6_) | 1 mM | 180 mg |
| 4 | HEPES | 10 mM | 1906.0 mg |
| 5 | Magnesium chloride hexahydrate (MgCl_2_.6H_2_O) | 49 mM | 16.2 mg |
| 6 | Sodium chloride (NaCl) | 105.05 mM | 1108.0 mg |
| 7 | Potassium chloride (KCl) | 2.3 mM | 44.6 mg |
| 8 | Sodium bicarbonate (NaHCO_3_) | 25 mM | 168.0 mg |
| 9 | Disodium phosphate (Na_2_HPO_4_) | 0.30 mM | 8.48 mg |
| 10 | Bovine serum albumin (BSA) | 3 mg/ml | 600.0 mg |
