# Supplementary material for: In vitro impact of ethanolic extract of Bryonia laciniosa seed on Gir bull spermatozoa: a comprehensive evaluation through transcriptome profiling
Source: Front Vet Sci. 2024 Jul 12;11:1419573. doi: 10.3389/fvets.2024.1419573 (PMC11273328; doi:10.3389/fvets.2024.1419573)
Supplement: Supplementary file 7 [file Table_2.docx]

**Table 2: Details of statistics pertaining mapped reads alignment data**

| **Sr. No.** | **Sample (spermatozoa)** | **No. of RNA-Seq reads**  **(millions)** | **Average length of reads**  **(bp)** | **Uniquely mapped reads**  **(Percentage)** | **Multiple mapped reads**  **(Percentage)** |
| --- | --- | --- | --- | --- | --- |
| 1 | Control motile | 2.243 | 411 | 65.80 | 33.19 |
| 2 | Control non-motile | 2.667 | 401 | 82.05 | 16.51 |
| 3 | Treated motile | 2.722 | 391 | 66.43 | 32.52 |
| 4 | Treated non-motile | 2.874 | 402 | 76.21 | 22.41 |
