# Supplementary material for: In vitro impact of ethanolic extract of Bryonia laciniosa seed on Gir bull spermatozoa: a comprehensive evaluation through transcriptome profiling
Source: Front Vet Sci. 2024 Jul 12;11:1419573. doi: 10.3389/fvets.2024.1419573 (PMC11273328; doi:10.3389/fvets.2024.1419573)
Supplement: Supplementary file 8 [file Table_3.docx]

**Table 3: Differential gene (top ten) expression between control motile and seed-S treated motile** **spermatozoa**

| **Gene** | **log2**  **(fold_change)** | **Gene Description and Function** |
| --- | --- | --- |
| *CRYGB* | 29.0284761 | Crystallin Gamma B. Crystallins are the dominant. It is structural components of the vertebrate eye lens |
| *KLF13* | 27.5331685 | Kruppel Like Factor 13. It represses transcription by binding to the BTE site, a GC-rich DNA element, in competition with the activator SP1 |
| *RPL32* | 27.0994483 | Ribosomal Protein L32 is structural constituent of ribosome |
| *STOML3* | 26.977361 | Stomatin Like 3. It required for the function of many mechanoreceptors |
| *CLDN15* | 26.4349221 | Claudin 15 is major constituents of the tight junction complexes that regulate the permeability of epithelia |
| *PRDX3* | 26.0960471 | Peroxiredoxin 3 has role in cell protection against oxidative stress by detoxifying peroxides |
| *SELENOM* | 26.019954 | Selenoprotein M. It function as a thiol-disulfide oxidoreductase that participates in disulfide bond formation |
| *GJC3* | 25.8260978 | Gap Junction Protein Gamma 3 has protein homodimerization activity |
| *TLX1* | 25.6882957 | T Cell Leukemia Homeobox 1 has controls the genesis of the spleen |
| *ZAR1* | 25.5867973 | Zygote Arrest 1. It play a role in the oocyte-to-embryo transition |
| Down regulated genes | | |
| *SLC25A42* | -24.67214 | Solute Carrier Family 25 Member 42. It has role in mitochondria in exchange for intramitochondrial (deoxy)adenine nucleotides and adenosine 3',5'-diphosphate. |
| *RUVBL2* | -24.6764885 | RuvB Like AAA ATPase 2. RNA polymerase II distal enhancer sequence-specific DNA binding |
| *CAMK2B* | -24.6929709 | Calcium/Calmodulin Dependent Protein Kinase II Beta. It involved in dendritic spine and synapse formation, neuronal plasticity and regulation of sarcoplasmic reticulum Ca(2+) transport in skeletal muscle |
| *MAPK1* | -24.8625046 | Mitogen-Activated Protein Kinase 1. It acts as an essential component of the MAP kinase signal transduction pathway |
| *KRTAP11-1* | -24.8640739 | Keratin Associated Protein 11-1 has role in the hair cortex, hair keratin intermediate filaments are embedded in an interfilamentous matrix |
| *INSM2* | -24.9823445 | INSM Transcriptional Repressor 2. It play role as a growth suppressor |
| *ARF4* | -25.3354293 | ADP Ribosylation Factor 4. It is an allosteric activator of the cholera toxin catalytic subunit, an ADP-ribosyltransferase |
| *NAGK* | -25.4628689 | N-Acetylglucosamine Kinase involved in the N-glycolylneuraminic acid (Neu5Gc) degradation pathway |
| *GPX6* | -25.4679914 | Glutathione Peroxidase 6 has glutathione peroxidase activity |
| *AQP7* | -25.4898791 | Aquaporin 7. It play role in body energy homeostasis under conditions that promote lipid catabolism, giving rise to glycerol and free fatty acids |
