# Supplementary material for: In vitro impact of ethanolic extract of Bryonia laciniosa seed on Gir bull spermatozoa: a comprehensive evaluation through transcriptome profiling
Source: Front Vet Sci. 2024 Jul 12;11:1419573. doi: 10.3389/fvets.2024.1419573 (PMC11273328; doi:10.3389/fvets.2024.1419573)
Supplement: Supplementary file 9 [file Table_4.docx]

**Table 4: Differential gene (top ten) expression between control non-motile and seed-S treated** **non-motile** **spermatozoa**

| **Gene** | **log2**  **(fold_change)** | **Gene Description and Function** |
| --- | --- | --- |
| *ZNF576* | 27.1368144 | Zinc Finger Protein 576 involved in transcriptional regulation |
| *TMEM42* | 26.5217798 | Transmembrane protein 42 has highest expression level in testis |
| *MTX2* | 26.3112398 | Metaxin 2 involved in transport of proteins into the mitochondrion |
| *FBXL12* | 25.9272792 | F-Box and Leucine Rich Repeat Protein 12 has ubiquitin protein ligase activity |
| *CLEC11A* | 25.9074876 | C-Type Lectin Domain Containing 11A. It promotes osteogenesis by stimulating the differentiation of mesenchymal progenitors into mature osteoblasts |
| *ZNF821* | 25.8533619 | Zinc Finger Protein 821 involved in transcriptional regulation |
| *CCS* | 25.8098623 | Copper Chaperone for Superoxide Dismutase. It delivers copper to copper zinc superoxide dismutase |
| *C13H20orf24* | 25.6763298 | Bos taurus RAB5-interacting protein (*C13H20orf24*), mRNA |
| *PRSS57* | 25.6100997 | Serine Protease 57. Serine protease that cleaves preferentially after Arg residues |
| *KCNK12* | 25.5888484 | Potassium Two Pore Domain Channel Subfamily K Member 12. It is probable potassium channel subunit. No channel activity observed in heterologous systems |
| Down regulated genes | | |
| *OIP5* | -25.1840183 | Opa Interacting Protein 5 required for recruitment of CENPA to centromeres and normal chromosome segregation during mitosis |
| *XKRX* | -25.2097318 | XK Related X-Linked. It has involvement of Increased transferrin (TF) endocytosis |
| *TAGLN2* | -25.2501963 | Transgelin 2, one of the earliest markers of differentiated smooth muscle cells and adult tissues,murine 2,Sm22 alpha homolog |
| *HCCS* | -25.2554862 | Holocytochrome C Synthase is holocytochrome C-type synthetase, involved in electron transport pathway |
| *GALNT3* | -25.3164197 | Polypeptide N-Acetylgalactosaminyltransferase 3. It catalyzes the initial reaction in O-linked oligosaccharide biosynthesis |
| *ING5* | -25.364371 | Inhibitor of Growth Family Member 5 is responsible for the bulk of histone H4 acetylation *in vivo* |
| *STUB1* | -25.4396105 | STIP1 Homology and U-Box Containing Protein 1  targets misfolded chaperone substrates towards proteasomal degradation |
| *TSPAN31* | -25.5724266 | Tetraspanin 31 involved in cell-surface proteins, growth-related cellular processes |
| *CCL25* | -25.8286541 | C-C Motif Chemokine Ligand 25. It is potentially involved in T-cell development |
| *ESX1* | -25.86815 | ESX Homeobox 1. It is coordinately regulate cell cycle progression and transcription during spermatogenesis |
